# Synthesis, crystal structure and thermal properties of bis­(aceto­nitrile-κ*N*)bis­(3-bromo­pyridine-κ*N*)bis­(thio­cyanato-κ*N*)cobalt(II)

**DOI:** 10.1107/S2056989022011380

**Published:** 2023-01-01

**Authors:** Christoph Krebs, Inke Jess, Christian Näther

**Affiliations:** a Institute of Inorganic Chemistry, University of Kiel, Max-Eyth-Str. 2, 24118 Kiel, Germany; University of Aberdeen, United Kingdom

**Keywords:** crystal structure, cobalt(II)thio­cyanate, 3-bromo­pyridine, thermal properties

## Abstract

Single crystals of the title compound, [Co(NCS)_2_(C_5_H_4_BrN)_2_(C_2_H_3_N)_2_], were obtained by the reaction of Co(NCS)_2_ with 3-bromo­pyridine in aceto­nitrile. The Co^II^ cations lie on crystallographic inversion centers and are coordinated by two N-bonded thio­cyanate anions, two 3-bromo­pyridine and two aceto­nitrile ligands thereby forming slightly distorted CoN_6_ octa­hedra. In the crystal, these complexes are linked by C—H⋯S and C—H⋯N hydrogen bonds into a three-dimensional network. In the direction of the crystallographic *b*-axis, the complexes are arranged into columns with neighboring 3-bromo­pyridine ligands stacked onto each other, indicating π–π inter­actions. The CN stretching vibration of the thio­cyanate anions is observed at 2066 cm^−1^, in agreement with the presence of only N-bonded anionic ligands. TG-DTA measurements reveal that in the first mass loss the aceto­nitrile ligands are removed and that in the second step, half of a 3-bromo­pyridine ligand is lost, leading to the formation of a polymeric compound with the composition [(Co(NCS)_2_)_2_(C_5_H_4_BrN)_3_]_*n*_ already reported in the literature.

## Abstract 2

The crystal structure of the title compound consists of discrete octa­hedral complexes, which are linked by inter­molecular C—H⋯S and C—H⋯N hydrogen bonds into chains.

## 1. Chemical context

Coordination compounds based on thio­cyanate anions show a large structural variability, which to some extend can be traced back to the versatile coordination behavior of this ligand and this is surely one reason why, for example, many isomeric compounds are known (Jochim *et al.*, 2020*a* ▸; Böhme *et al.*, 2020 ▸; Neumann *et al.*, 2018 ▸; Werner *et al.*, 2015*a* ▸). Moreover, in bridging thio­cyanate anions a reasonable magnetic exchange is present, which can lead to compounds with a variety of magnetic properties (Palion-Gazda *et al.*, 2015 ▸; Mekuimemba *et al.*, 2018 ▸). In this context, compounds based on Co^II^ cations are of special inter­est, not only because they can show anti­ferromagnetic or ferromagnetic ordering but as also for the strong magnetic anisotropy and slow relaxation of the magnetization indicative of single-chain-magnet behavior that can be observed (Böhme *et al.*, 2019 ▸; Switlicka *et al.*, 2020 ▸; Werner *et al.*, 2014 ▸; Shurdha *et al.*, 2013 ▸; Prananto *et al.*, 2017 ▸; Mautner *et al.*, 2018 ▸). The latter is observable in linear chain compounds, in which the Co^II^ cations are octa­hedrally coord­inated in an all *trans*-configuration and linked by pairs of thio­cyanate anions (Werner *et al.*, 2015*b* ▸; Mautner *et al.*, 2018 ▸; Rams *et al.*, 2020 ▸). This is the most common structural motif for such compounds, although corrugated chains or layers are also known (Chen *et al.*, 2002 ▸; Wang *et al.*, 2005 ▸; Jin *et al.*, 2007 ▸; Shi *et al.*, 2007 ▸; Yang *et al.*, 2007 ▸; Suckert *et al.*, 2016 ▸). All of these are reasons why we became inter­ested in this class of compounds in order to study the influence of a chemical and a structural modification on their magnetic properties (Wöhlert *et al.*, 2014 ▸; Neumann *et al.*, 2019 ▸; Rams *et al.*, 2017*a* ▸,*b* ▸, 2020 ▸; Jochim *et al.*, 2020*b* ▸; Ceglarska *et al.*, 2021 ▸).

In this context we have reported on the synthesis, crystal structures and magnetic properties of coordination compounds based on Co(NCS)_2_ and 3-bromo­pyridine (C_5_H_4_BrN) as a ligand (Böhme *et al.*, 2022 ▸). During these investigations, we obtained discrete complexes with the composition Co(NCS)_2_(3-bromo­pyridine)_4_, Co(NCS)_2_(3-bromo­pyridine)_2_(H_2_O)_2_ and Co(NCS)_2_(3-bromo­pyri­dine)_2_(MeOH)_2_ that lose the ligands stepwise upon heating, leading to the formation of compounds with the composition [(Co(NCS)_2_)_2_(3-bromo­pyridine)_3_]_*n*_ and [Co(NCS)_2_(3-bromo­pyridine)_2_]_*n*_, in which the Co cations are linked into chains by pairs of *μ*-1,3 bridging thio­cyanate anions. In the latter compound, each of the Co^II^ cations is octa­hedrally coordinated, whereas in the former an alternating octa­hedral and square-planar coordination is observed, which is very rare for thio­cyanate compounds and had never been observed previously with Co(NCS)_2_. Later it was found that the compound with the mixed coordination can also be obtained from solution, which is impossible for the other compound with only an octa­hedral coordination. Unfortunately, the synthesis of the latter is difficult to achieve because all thermogravimetric curves are not well resolved and thermal annealing of the discrete complexes can lead to pure samples, but sometimes this is not the case. Therefore, we looked for other precursors that might show a similar reactivity and that might lead more easily to pure samples. In the course of these investigations, we obtained crystals of the title compound by the reaction of Co(NCS)_2_, 3-bromo­pyridine and aceto­nitrile. The CN stretching vibration of the thio­cyanate anions is observed at 2066 cm^−1^ in the IR spectrum, which points to the presence of terminal N-bonded thio­cyanate anions (Fig. S1). Single-crystal structure analysis proved that the structure consists of discrete complexes with the composition Co(NCS)_2_(3-bromo­pyridine)_2_(aceto­nitrile)_2_ and a comparison of the experimental XRPD pattern with that calculated from single-crystal data reveals that a pure phase has been obtained (Fig. S2). Therefore, this compound might be a suitable precursor for the synthesis of compounds, in which the Co^II^ cations are linked by *μ*-1,3 bridging thio­cyanate anions into chains. 
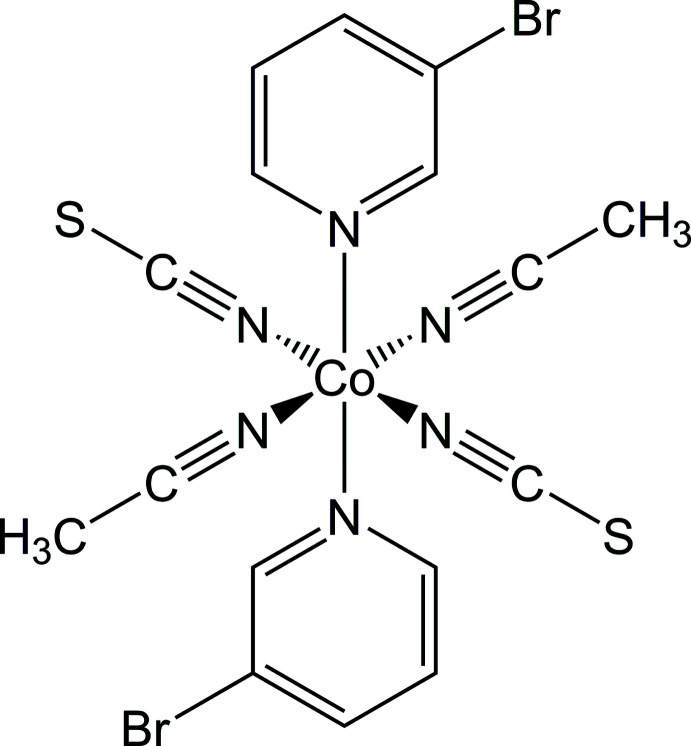


Investigations using thermogravimetry and differential thermoanalysis (TG-DTA) show several mass losses, that are each accompanied with endothermic events in the DTA curve (Fig. S3). The experimental mass loss is in excellent agreement with that, calculated for the removal of two aceto­nitrile ligands (Δ*m* = 14.3%), whereas the values for the second and third mass loss roughly correspond to the emission of half a 3-bromo­pyridine ligand in each step (Δ*m* = 13.8%). Therefore, one can assume that in the first TG step a compound with the composition Co(NCS)_2_(3-bromo­pyridine)_2_ will form, which transforms into (Co(NCS)_2_)_2_(3-bromo­pyridine)_3_ upon further heating. XRPD investigations of the residue obtained after the first mass loss reveal the formation of a compound of poor crystallinity that cannot be identified. In contrast, a comparison of the powder pattern of the residue formed after the second TG step with that calculated for [(Co(NCS)_2_)_2_(3-bromo­pyridine)_3_]_*n*_ (Böhme *et al.*, 2022 ▸) retrieved from the literature proves that this chain compound has formed (Fig. S4).

## 2. Structural commentary

**Figure 1:**
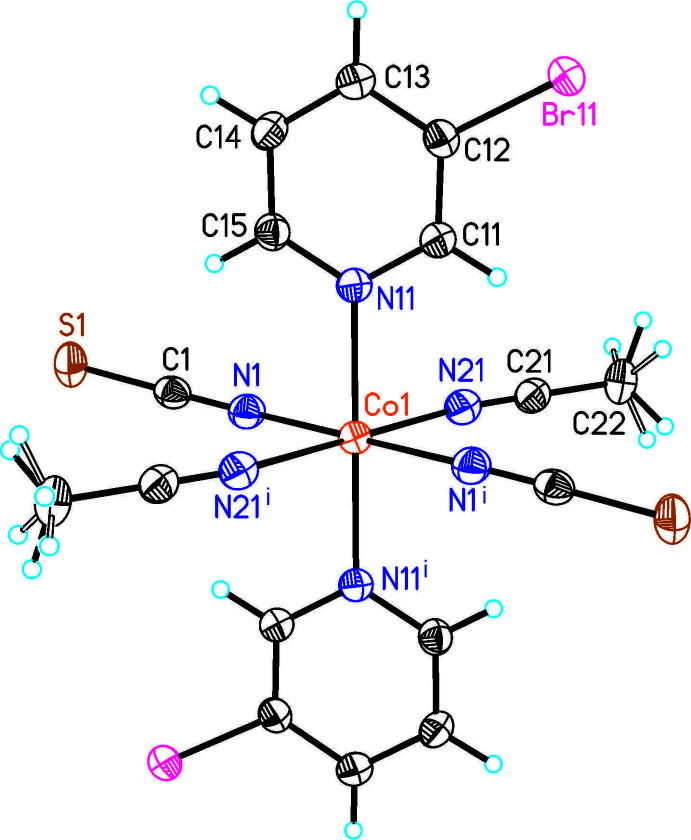
Crystal structure of the title compound with atom labeling and displacement ellipsoids drawn at the 50% probability level. Symmetry code: (i) −*x* + 1, −*y* + 1, −*z* + 1. The disorder of the methyl H atoms is shown as full and open bonds.

The asymmetric unit of the title compound consists of one crystallographically independent Co cation that is located on a center of inversion, as well as one thio­cyanate anion, one 3-bromo­pyridine and one aceto­nitrile ligand in general positions (Fig. 1 ▸). The methyl H atoms of the aceto­nitrile ligands are disordered over two orientations rotated by about 120° and were refined using a split model. The Co^II^ cations are octa­hedrally coordinated by two symmetry-related 3-bromo­pyridine and two aceto­nitrile ligands as well as two terminal N-bonded thio­cyanate anions into discrete complexes (Fig. 1 ▸). Bond lengths and angles correspond to literature values and from the bonding angles it is obvious that the octa­hedra are moderately distorted (Fig. 1 ▸ and Table 1 ▸). This is in agreement with the values for the octa­hedral angle variance of 10.02°^2^ and the mean octa­hedral quadratic elongation of 1.0037, calculated using the method of Robinson *et al.* (1971 ▸). The six-membered rings of the 3-bromo­pyridine ligands coordinating to the Co^II^ cations are coplanar by symmetry.

## 3. Supra­molecular features

**Figure 2:**
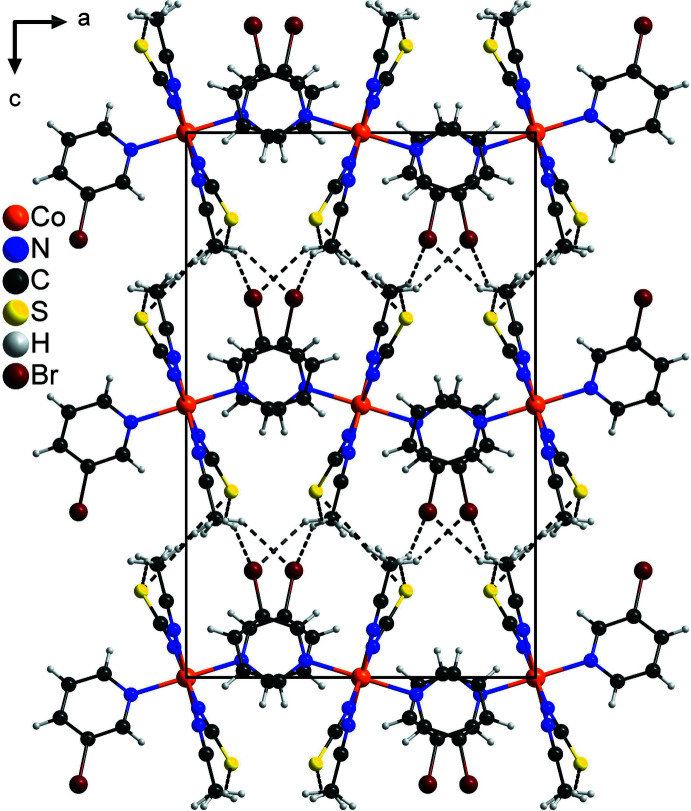
Crystal structure of the title compound with view along the crystallographic *b*-axis and inter­molecular C—H⋯S and C—H⋯Br hydrogen bonding shown as dashed lines. Please note that the methyl H atoms of the aceto­nitrile ligand are disordered.

**Figure 3:**
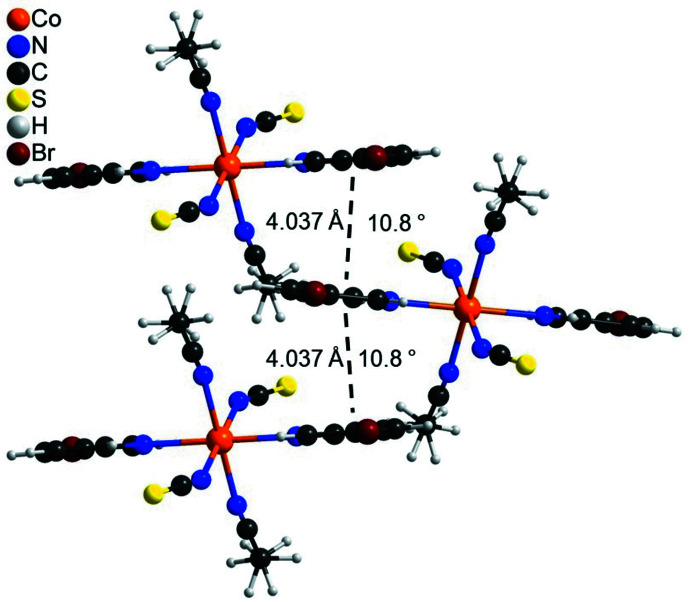
Crystal structure of the title compound showing the orientation of neighboring 3-bromo­pyridine rings. The distance between the centroids of the six-membered rings and the angles between the ring planes are shown. Please note that the methyl H atoms of the aceto­nitrile ligand are disordered.

In the extended structure of the title compound, the discrete complexes are linked by C—H⋯S and C—H⋯Br inter­actions into a three-dimensional network (Fig. 2 ▸ and Table 2 ▸). One C—H⋯S angle is close to linear, whereas the other C—H⋯S and C—H⋯Br angles are much less than 180°, indicating only weak inter­actions (Table 2 ▸). There are additional C—H⋯N contacts but their bonding angles are very far from linear (Table 2 ▸). The discrete complexes are arranged in stacks that propagate along the crystallographic *b*-axis direction (Fig. 2 ▸). Within these stacks, neighboring pyridine rings are nearly coplanar with an angle between their mean planes of 10.8 (1)° and a distance between the centroids of the rings of 4.037 (1) Å, indicating very weak π–π stacking inter­actions (Fig. 3 ▸).

## 4. Database survey

Some crystal structures with thio­cyanate anions and 3-bromo­pyridine as a coligand have already been reported in the Cambridge Structural Database (CSD version 5.42, last update November 2021; Groom *et al.*, 2016 ▸). They include [Cu(NCS)_2_(3-bromo­pyridine)_2_]_2_, which consists of dimers, in which each Cu^II^ cation is coordinated by two 3-bromo­pyridine coligands as well as one terminal and two *μ*-1,3 bridging thio­cyanate anions (Handy *et al.*, 2017 ▸). In CuNCS(3-bromo­pyridine), the copper(I) cations are tetra­hedrally coordinated and linked into layers by *μ*-1,3 bridging thio­cyanate anions (Miller *et al.*, 2011 ▸). In the second Cu^II^ compound (CuNCS)_2_(3-bromo­pyridine)_4_, the copper cations are also tetra­hedrally coordinated but linked into chains by *μ*-1,3 bridging thio­cyanate anions (Nicholas, *et al.*, 2017 ▸).

With Ni(NCS)_2_ and 3-bromo­pyridine several compounds are reported, including the discrete complexes with octa­hedrally coordinated Ni^II^ cations Ni(NCS)_2_(3-bromo­pyridine)_4_, Ni(NCS)_2_(3-bromo­pyridine)_2_(H_2_O)_2_ and Ni(NCS)_2_(3-bromo­pyridine)_2_(CH_3_OH)_2_ (Krebs *et al.*, 2021 ▸). Also included is a compound with aceto­nitrile with the composition Ni(NCS)_2_(3-bromo­pyridine)_2_·CH_3_CN, but in this structure the Ni^II^ cations are linked into corrugated chains by *μ*-1,3 bridging thio­cyanate anions that are connected *via* inter­molecular hydrogen bonding into a three-dimensional network that contains channels in which aceto­nitrile solvate mol­ecules are embedded (Krebs *et al.*, 2021 ▸). Finally, when the discrete complexes are heated, a transformation into Ni(NCS)_2_(3-bromo­pyridine)_2_ is observed, in which the Ni^II^ cations are octa­hedrally coordinated and linked into chains by pairs of *μ*-1,3 bridging thio­cyanate anions (Krebs *et al.*, 2021 ▸). The latter compound is isotypic to its Co^II^ analog reported recently (Böhme *et al.*, 2022 ▸).

With diamagnetic cations, four structures are reported. The compounds *M*(NCS)_2_(3-bromo­pyridine)_4_ (*M* = Zn, Cd) are isotypic and consist of discrete complexes in which the metal cations are octa­hedrally coordinated by four 3-bromo­pyridine ligands and two terminal N-bonded thio­cyanate anions (Wöhlert *et al.*, 2013 ▸). Upon heating, half of the 3-bromo­pyridine ligands are removed and a transformation into compounds of the composition *M*(NCS)_2_(3-bromo­pyridine)_2_ (*M* = Zn, Cd) is observed (Wöhlert *et al.*, 2013 ▸). The Zn compounds consist of tetra­hedral complexes, whereas in the Cd compound the cations are linked by pairs of anionic ligands into chains.

## 5. Synthesis and crystallization

Co(NCS)_2_ and 3-bromo­pyridine were purchased from Merck. All chemicals were used without further purification. After storing 0.5 mmol of Co(NCS)_2_ (87.5 mg) and 0.5 mmol of 3-bromo­pyridine (48.8 µl) in 2.0 ml of aceto­nitrile for 3 d at room temperature, light-red single crystals of the title compound suitable for single-crystal X-ray analysis were obtained. The IR spectrum was measured using an ATI Mattson Genesis Series FTIR Spectrometer, control software *WINFIRST*, from ATI Mattson. The XRPD measurement was performed with Cu *K*α_1_ radiation (λ = 1.540598 Å) using a Stoe Transmission Powder Diffraction System (STADI P) equipped with a MYTHEN 1K detector and a Johansson-type Ge(111) monochromator. Thermogravimetry and differential thermoanalysis (TG–DTA) measurements were performed in a dynamic nitrogen atmosphere in Al_2_O_3_ crucibles using a STA-PT 1000 thermobalance from Linseis. The instrument was calibrated using standard reference materials.

## 6. Refinement

The C-bound H atoms were positioned with idealized geometry (methyl H atoms allowed to rotate but not to tip) and were refined isotropically with *U*_iso_(H) = 1.2*U*_eq_(C) (1.5 for methyl H atoms) using a riding model. The H atoms of the methyl group of the aceto­nitrile ligand are disordered in two orientations and were refined in ratio 50:50 using a split model. Crystal data, data collection and structure refinement details are summarized in Table 3 ▸.

## Supplementary Material

Crystal structure: contains datablock(s) I. DOI: 10.1107/S2056989022011380/hb8045sup1.cif

Structure factors: contains datablock(s) I. DOI: 10.1107/S2056989022011380/hb8045Isup2.hkl

IR spectrum of the title compound. The value of the CN stretching vibration is given. DOI: 10.1107/S2056989022011380/hb8045sup3.png

Experimental (top) and calculated X-ray powder pattern (bottom) of the title compound. DOI: 10.1107/S2056989022011380/hb8045sup4.png

DTG (top) TG (mid) and DTA (bottom) curve of the title compound measured with 4C/min. The mass loss in % and the peak temperatures are given. DOI: 10.1107/S2056989022011380/hb8045sup5.png

Experimental powder pattern of the residue formed after the second TG step (top) and calculated powder pattern of [(Co(NCS)2)2(3-bromopyridine)3]n retrieved from the literature (bottom). DOI: 10.1107/S2056989022011380/hb8045sup6.png

CCDC reference: 2222142

Additional supporting information:  crystallographic information; 3D view; checkCIF report

## Figures and Tables

**Table 1:** Selected geometric parameters (Å, °)

Co1—N1	2.0736 (19)	Co1—N21	2.161 (2)
Co1—N11	2.1759 (19)		
			
N1^i^—Co1—N11	90.27 (7)	N21^i^—Co1—N11	90.59 (7)
N1—Co1—N11	89.73 (7)	N21—Co1—N11	89.41 (7)
N1^i^—Co1—N21	84.79 (7)	Co1—N1—C1	154.96 (18)
N1—Co1—N21	95.21 (7)	Co1—N21—C21	160.05 (18)

Symmetry code: (i) 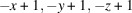 .

**Table 2:** Hydrogen-bond geometry (Å, °)

*D*—H⋯*A*	*D*—H	H⋯*A*	*D*⋯*A*	*D*—H⋯*A*
C11—H11⋯N1^i^	0.95	2.60	3.109 (3)	114
C15—H15⋯N1	0.95	2.63	3.121 (3)	113
C22—H22*A*⋯S1^ii^	0.98	2.98	3.947 (3)	168
C22—H22*B*⋯S1^iii^	0.98	2.76	3.625 (3)	147
C22—H22*C*⋯Br11^iv^	0.98	2.97	3.840 (3)	148
C22—H22*F*⋯Br11^v^	0.98	2.92	3.681 (3)	135

Symmetry codes: (i) 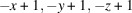 ; (ii) 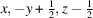 ; (iii) 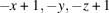 ; (iv) 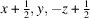 ; (v) 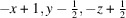 .

**Table 3:** Experimental details

Crystal data	
Chemical formula	[Co(NCS)_2_(C_5_H_4_BrN)_2_(C_2_H_3_N)_2_]
*M*_r_	573.20
Crystal system, space group	Orthorhombic, *P* *b* *c* *a*
Temperature (K)	100
*a*, *b*, *c* (Å)	13.1206 (2), 8.0606 (2), 20.4520 (4)
*V* (Å^3^)	2163.00 (8)
*Z*	4
Radiation type	Cu *K*α
μ (mm^−1^)	12.47
Crystal size (mm)	0.16 × 0.08 × 0.02

Data collection	
Diffractometer	XtaLAB Synergy, Dualflex, HyPix
Absorption correction	Multi-scan (*CrysAlis PRO*; Rigaku OD, 2021 ▸)
*T*_min_, *T*_max_	0.689, 1.000
No. of measured, independent and observed [*I* > 2σ(*I*)] reflections	10492, 2289, 2142
*R*_int_	0.023
(sin θ/λ)_max_ (Å^−1^)	0.634

Refinement	
*R*[*F* ^2^ > 2σ(*F* ^2^)], *wR*(*F* ^2^), *S*	0.027, 0.084, 1.10
No. of reflections	2289
No. of parameters	126
H-atom treatment	H-atom parameters constrained
Δρ_max_, Δρ_min_ (e Å^−3^)	0.38, −0.53

Computer programs: *CrysAlis PRO* (Rigaku OD, 2021 ▸), *SHELXT2014/5* (Sheldrick, 2015*a* ▸), *SHELXL2016/6* (Sheldrick, 2015*b* ▸), *DIAMOND* (Brandenburg & Putz, 1999 ▸) and *publCIF* (Westrip, 2010 ▸).

## supplementary crystallographic information

### Crystal data

**Table:**

[Co(NCS)_2_(C_5_H_4_BrN)_2_(C_2_H_3_N)_2_]	*D*_x_ = 1.760 Mg m^−^^3^
*M**_r_* = 573.20	Cu *K*α radiation, λ = 1.54184 Å
Orthorhombic, *P**b**c**a*	Cell parameters from 8002 reflections
*a* = 13.1206 (2) Å	θ = 4.3–77.9°
*b* = 8.0606 (2) Å	µ = 12.47 mm^−^^1^
*c* = 20.4520 (4) Å	*T* = 100 K
*V* = 2163.00 (8) Å^3^	Block, light red
*Z* = 4	0.16 × 0.08 × 0.02 mm
*F*(000) = 1124	

### Data collection

**Table:**

XtaLAB Synergy, Dualflex, HyPix diffractometer	2289 independent reflections
Radiation source: micro-focus sealed X-ray tube, PhotonJet (Cu) X-ray Source	2142 reflections with *I* > 2σ(*I*)
Mirror monochromator	*R*_int_ = 0.023
Detector resolution: 10.0000 pixels mm^-1^	θ_max_ = 77.9°, θ_min_ = 4.3°
ω scans	*h* = −16→13
Absorption correction: multi-scan (CrysalisPro; Rigaku OD, 2021)	*k* = −9→10
*T*_min_ = 0.689, *T*_max_ = 1.000	*l* = −25→24
10492 measured reflections	

### Refinement

**Table:**

Refinement on *F*^2^	Hydrogen site location: inferred from neighbouring sites
Least-squares matrix: full	H-atom parameters constrained
*R*[*F*^2^ > 2σ(*F*^2^)] = 0.027	*w* = 1/[σ^2^(*F*_o_^2^) + (0.0583*P*)^2^ + 0.8249*P*] where *P* = (*F*_o_^2^ + 2*F*_c_^2^)/3
*wR*(*F*^2^) = 0.084	(Δ/σ)_max_ = 0.001
*S* = 1.10	Δρ_max_ = 0.38 e Å^−^^3^
2289 reflections	Δρ_min_ = −0.53 e Å^−^^3^
126 parameters	Extinction correction: SHELXL2016/6 (Sheldrick 2015b), Fc^*^=kFc[1+0.001xFc^2^λ^3^/sin(2θ)]^-1/4^
0 restraints	Extinction coefficient: 0.00076 (10)
Primary atom site location: dual	

### Special details

**Table:**

Geometry. All esds (except the esd in the dihedral angle between two l.s. planes) are estimated using the full covariance matrix. The cell esds are taken into account individually in the estimation of esds in distances, angles and torsion angles; correlations between esds in cell parameters are only used when they are defined by crystal symmetry. An approximate (isotropic) treatment of cell esds is used for estimating esds involving l.s. planes.

### Fractional atomic coordinates and isotropic or equivalent isotropic displacement parameters (Å^2^)

**Table:**

	*x*	*y*	*z*	*U*_iso_*/*U*_eq_	Occ. (<1)
Co1	0.500000	0.500000	0.500000	0.02317 (15)	
N1	0.46546 (15)	0.2934 (2)	0.55618 (9)	0.0277 (4)	
C1	0.42663 (16)	0.2229 (3)	0.59925 (10)	0.0261 (4)	
S1	0.37115 (5)	0.12261 (8)	0.65871 (3)	0.03667 (17)	
N11	0.34175 (14)	0.5136 (2)	0.46864 (9)	0.0243 (4)	
C11	0.31849 (16)	0.5744 (3)	0.40943 (11)	0.0258 (4)	
H11	0.371856	0.612144	0.381775	0.031*	
C12	0.21868 (18)	0.5837 (3)	0.38751 (10)	0.0252 (4)	
C13	0.13924 (17)	0.5257 (3)	0.42614 (11)	0.0265 (4)	
H13	0.070717	0.528518	0.411177	0.032*	
C14	0.16372 (18)	0.4638 (3)	0.48723 (12)	0.0271 (4)	
H14	0.111742	0.423819	0.515468	0.033*	
C15	0.26519 (17)	0.4606 (3)	0.50692 (10)	0.0256 (4)	
H15	0.280996	0.419248	0.549211	0.031*	
Br11	0.19160 (2)	0.67390 (3)	0.30387 (2)	0.02899 (12)	
N21	0.53585 (15)	0.3613 (2)	0.41254 (10)	0.0289 (4)	
C21	0.55714 (17)	0.3339 (3)	0.35970 (12)	0.0275 (5)	
C22	0.5843 (2)	0.3030 (3)	0.29171 (13)	0.0364 (5)	
H22A	0.523662	0.315482	0.264145	0.055*	0.5
H22B	0.610963	0.189959	0.287354	0.055*	0.5
H22C	0.636420	0.382640	0.277910	0.055*	0.5
H22D	0.550407	0.384647	0.263646	0.055*	0.5
H22E	0.562352	0.191093	0.279293	0.055*	0.5
H22F	0.658286	0.312341	0.286471	0.055*	0.5

### Atomic displacement parameters (Å^2^)

**Table:**

	*U*^11^	*U*^22^	*U*^33^	*U*^12^	*U*^13^	*U*^23^
Co1	0.0217 (3)	0.0256 (3)	0.0222 (3)	−0.00087 (18)	−0.00012 (17)	0.00064 (18)
N1	0.0256 (9)	0.0280 (9)	0.0294 (9)	−0.0008 (7)	−0.0015 (7)	0.0028 (7)
C1	0.0233 (9)	0.0283 (10)	0.0268 (10)	0.0033 (8)	−0.0026 (8)	−0.0037 (9)
S1	0.0440 (3)	0.0407 (3)	0.0253 (3)	−0.0020 (3)	0.0058 (2)	0.0034 (2)
N11	0.0224 (8)	0.0268 (8)	0.0237 (9)	−0.0009 (7)	−0.0001 (7)	−0.0009 (7)
C11	0.0243 (10)	0.0285 (10)	0.0247 (10)	−0.0022 (8)	0.0002 (8)	−0.0002 (9)
C12	0.0285 (10)	0.0244 (9)	0.0225 (9)	−0.0006 (8)	−0.0011 (8)	−0.0001 (8)
C13	0.0235 (10)	0.0271 (10)	0.0289 (11)	−0.0004 (8)	−0.0001 (8)	0.0000 (8)
C14	0.0235 (10)	0.0285 (10)	0.0293 (10)	−0.0004 (9)	0.0048 (9)	0.0028 (9)
C15	0.0261 (11)	0.0273 (10)	0.0233 (10)	0.0018 (9)	0.0009 (8)	0.0001 (8)
Br11	0.02896 (17)	0.03464 (17)	0.02338 (17)	−0.00212 (9)	−0.00260 (7)	0.00317 (8)
N21	0.0259 (9)	0.0313 (9)	0.0293 (9)	−0.0008 (7)	0.0006 (8)	−0.0004 (8)
C21	0.0251 (10)	0.0270 (10)	0.0305 (12)	−0.0004 (8)	0.0011 (9)	0.0025 (8)
C22	0.0416 (14)	0.0391 (12)	0.0283 (11)	−0.0008 (11)	0.0092 (10)	0.0035 (10)

### Geometric parameters (Å, º)

**Table:**

Co1—N1^i^	2.0736 (19)	C13—H13	0.9500
Co1—N1	2.0736 (19)	C13—C14	1.383 (3)
Co1—N11	2.1759 (19)	C14—H14	0.9500
Co1—N11^i^	2.1758 (19)	C14—C15	1.391 (3)
Co1—N21^i^	2.161 (2)	C15—H15	0.9500
Co1—N21	2.161 (2)	N21—C21	1.138 (3)
N1—C1	1.165 (3)	C21—C22	1.457 (3)
C1—S1	1.632 (2)	C22—H22A	0.9800
N11—C11	1.342 (3)	C22—H22B	0.9800
N11—C15	1.343 (3)	C22—H22C	0.9800
C11—H11	0.9500	C22—H22D	0.9800
C11—C12	1.386 (3)	C22—H22E	0.9800
C12—C13	1.389 (3)	C22—H22F	0.9800
C12—Br11	1.892 (2)		
			
N1^i^—Co1—N1	180.0	C13—C12—Br11	120.17 (17)
N1^i^—Co1—N11	90.27 (7)	C12—C13—H13	121.3
N1—Co1—N11	89.73 (7)	C14—C13—C12	117.5 (2)
N1—Co1—N11^i^	90.27 (7)	C14—C13—H13	121.3
N1^i^—Co1—N11^i^	89.73 (7)	C13—C14—H14	120.3
N1^i^—Co1—N21	84.79 (7)	C13—C14—C15	119.4 (2)
N1—Co1—N21	95.21 (7)	C15—C14—H14	120.3
N1—Co1—N21^i^	84.79 (7)	N11—C15—C14	122.8 (2)
N1^i^—Co1—N21^i^	95.21 (7)	N11—C15—H15	118.6
N11^i^—Co1—N11	180.0	C14—C15—H15	118.6
N21^i^—Co1—N11^i^	89.42 (7)	Co1—N21—C21	160.05 (18)
N21^i^—Co1—N11	90.59 (7)	N21—C21—C22	178.7 (2)
N21—Co1—N11	89.41 (7)	C21—C22—H22A	109.5
N21—Co1—N11^i^	90.58 (7)	C21—C22—H22B	109.5
N21—Co1—N21^i^	180.00 (6)	C21—C22—H22C	109.5
Co1—N1—C1	154.96 (18)	C21—C22—H22D	109.5
N1—C1—S1	179.1 (2)	C21—C22—H22E	109.5
C11—N11—Co1	120.11 (14)	C21—C22—H22F	109.5
C11—N11—C15	118.17 (19)	H22A—C22—H22B	109.5
C15—N11—Co1	121.72 (15)	H22A—C22—H22C	109.5
N11—C11—H11	119.1	H22B—C22—H22C	109.5
N11—C11—C12	121.8 (2)	H22D—C22—H22E	109.5
C12—C11—H11	119.1	H22D—C22—H22F	109.5
C11—C12—C13	120.5 (2)	H22E—C22—H22F	109.5
C11—C12—Br11	119.37 (17)		

Symmetry code: (i) −*x*+1, −*y*+1, −*z*+1.

### Hydrogen-bond geometry (Å, º)

**Table:**

*D*—H···*A*	*D*—H	H···*A*	*D*···*A*	*D*—H···*A*
C11—H11···N1^i^	0.95	2.60	3.109 (3)	114
C15—H15···N1	0.95	2.63	3.121 (3)	113
C22—H22*A*···S1^ii^	0.98	2.98	3.947 (3)	168
C22—H22*B*···S1^iii^	0.98	2.76	3.625 (3)	147
C22—H22*C*···Br11^iv^	0.98	2.97	3.840 (3)	148
C22—H22*F*···Br11^v^	0.98	2.92	3.681 (3)	135

Symmetry codes: (i) −*x*+1, −*y*+1, −*z*+1; (ii) *x*, −*y*+1/2, *z*−1/2; (iii) −*x*+1, −*y*, −*z*+1; (iv) *x*+1/2, *y*, −*z*+1/2; (v) −*x*+1, *y*−1/2, −*z*+1/2.

## References

[bb1] Böhme, M., Jochim, A., Rams, M., Lohmiller, T., Suckert, S., Schnegg, A., Plass, W. & Näther, C. (2020). *Inorg. Chem.* **59**, 5325–5338. 10.1021/acs.inorgchem.9b03357 32091883

[bb2] Böhme, M. & Plass, W. (2019). *Chem. Sci.* **10**, 9189–9202. 10.1039/c9sc02735a PMC6979495 32055306

[bb3] Böhme, M., Rams, M., Krebs, C., Mangelsen, S., Jess, I., Plass, W. & Näther, C. (2022). *Inorg. Chem.* **61**, 16841–16855. 10.1021/acs.inorgchem.2c02813 36218356

[bb4] Brandenburg, K. & Putz, H. (1999). *DIAMOND*. Crystal Impact GbR, Bonn, Germany.

[bb5] Ceglarska, M., Böhme, M., Neumann, T., Plass, W., Näther, C. & Rams, M. (2021). *Phys. Chem. Chem. Phys.* **23**, 10281–10289. 10.1039/d1cp00719j 33903874

[bb6] Chen, H. & Chen, X. M. (2002). *Inorg. Chim. Acta*, **329**, 13–21.

[bb7] Groom, C. R., Bruno, I. J., Lightfoot, M. P. & Ward, S. C. (2016). *Acta Cryst.* B**72**, 171–179. 10.1107/S2052520616003954 PMC4822653 27048719

[bb8] Handy, J. V., Ayala, G. & Pike, R. D. (2017). *Inorg. Chim. Acta*, **456**, 64–75.

[bb9] Jin, Y., Che, Y. X. & Zheng, J. M. (2007). *J. Coord. Chem.* **60**, 2067–2074.

[bb10] Jochim, A., Lohmiller, T., Rams, M., Böhme, M., Ceglarska, M., Schnegg, A., Plass, W. & Näther, C. (2020*a*). *Inorg. Chem.* **59**, 8971–8982. 10.1021/acs.inorgchem.0c00815 32551545

[bb11] Jochim, A., Rams, M., Böhme, M., Ceglarska, M., Plass, W. & Näther, C. (2020*b*). *Dalton Trans.* **49**, 15310–15322. 10.1039/d0dt03227a 33118568

[bb12] Krebs, C., Ceglarska, M. & Näther, C. (2021). *Z. Anorg. Allg. Chem.* **647**, 552–559.

[bb13] Mautner, F. A., Traber, M., Fischer, R. C., Torvisco, A., Reichmann, K., Speed, S., Vicente, R. & Massoud, S. S. (2018). *Polyhedron*, **154**, 436–442.

[bb14] Mekuimemba, C. D., Conan, F., Mota, A. J., Palacios, M. A., Colacio, E. & Triki, S. (2018). *Inorg. Chem.* **57**, 2184–2192. 10.1021/acs.inorgchem.7b03082 29420016

[bb15] Miller, K. M., McCullough, S. M., Lepekhina, E. A., Thibau, I. J., Pike, R. D., Li, X., Killarney, J. P. & Patterson, H. H. (2011). *Inorg. Chem.* **50**, 7239–7249. 10.1021/ic200821f 21728324

[bb16] Neumann, C., Rams, M., Tomkowicz, Z., Jess, I. & Näther, C. (2019). *Chem. Commun.* **55**, 2652–2655. 10.1039/c8cc09392j 30742155

[bb17] Neumann, T., Ceglarska, M., Germann, L. S., Rams, M., Dinnebier, R. E., Suckert, S., Jess, I. & Näther, C. (2018). *Inorg. Chem.* **57**, 3305–3314. 10.1021/acs.inorgchem.8b00092 29505252

[bb18] Nicholas, A. D., Otten, B. M., Ayala, G., Hutchinson, J., Wojtas, L., Omary, M. A., Pike, R. D. & Patterson, H. H. (2017). *J. Phys. Chem.* **C121**, 25430–25439.

[bb19] Palion-Gazda, J., Machura, B., Lloret, F. & Julve, M. (2015). *Cryst. Growth Des.* **15**, 2380–2388.

[bb20] Prananto, Y. P., Urbatsch, A., Moubaraki, B., Murray, K. S., Turner, D. R., Deacon, G. B. & Batten, S. R. (2017). *Aust. J. Chem.* **70**, 516–528.

[bb21] Rams, M., Böhme, M., Kataev, V., Krupskaya, Y., Büchner, B., Plass, W., Neumann, T., Tomkowicz, Z. & Näther, C. (2017*b*). *Phys. Chem. Chem. Phys.* **19**, 24534–24544. 10.1039/c7cp04189f 28852749

[bb22] Rams, M., Jochim, A., Böhme, M., Lohmiller, T., Ceglarska, M., Rams, M. M., Schnegg, A., Plass, W. & Näther, C. (2020). *Chem. Eur. J.* **26**, 2837–2851. 10.1002/chem.201903924 PMC7078958 31702081

[bb23] Rams, M., Tomkowicz, Z. A., Böhme, M., Plass, W., Suckert, S., Werner, J., Jess, I. & Näther, C. (2017*a*). *Phys. Chem. Chem. Phys.* **19**, 3232–3243. 10.1039/c6cp08193b 28083584

[bb24] Rigaku OD (2021). *CrysAlis PRO*. Rigaku Oxford Diffraction.

[bb25] Robinson, K., Gibbs, G. V. & Ribbe, P. H. (1971). *Science*, **172**, 567–570. 10.1126/science.172.3983.567 17802221

[bb26] Sheldrick, G. M. (2015*a*). *Acta Cryst.* A**71**, 3–8.

[bb27] Sheldrick, G. M. (2015*b*). *Acta Cryst.* C**71**, 3–8.

[bb28] Shi, J. M., Chen, J. N., Wu, C. J. & Ma, J. P. (2007). *J. Coord. Chem.* **60**, 2009–2013.

[bb29] Shurdha, E., Moore, C. E., Rheingold, A. L., Lapidus, S. H., Stephens, P. W., Arif, A. M. & Miller, J. S. (2013). *Inorg. Chem.* **52**, 10583–10594. 10.1021/ic401558f 23981238

[bb30] Suckert, S., Rams, M., Böhme, M., Germann, L., Dinnebier, R. E., Plass, W., Werner, J. & Näther, C. (2016). *Dalton Trans.* **45**, 18190–18201. 10.1039/c6dt03752f 27796392

[bb31] Świtlicka, A., Machura, B., Kruszynski, R., Moliner, N., Carbonell, J. M., Cano, J., Lloret, F. & Julve, C. (2020). *Inorg. Chem. Front.* **7**, 4535–4552.

[bb32] Wang, X. Y., Li, B. L., Zhu, X. & Gao, S. (2005). *Eur. J. Inorg. Chem.* pp. 3277–3286.

[bb33] Werner, J., Rams, M., Tomkowicz, Z. & Näther, C. (2014). *Dalton Trans.* **43**, 17333–17342. 10.1039/c4dt02271h 25318637

[bb34] Werner, J., Runčevski, T., Dinnebier, R. E., Ebbinghaus, S. G., Suckert, S. & Näther, C. (2015*a*). *Eur. J. Inorg. Chem.* pp. 3236–3245. 10.1021/ic503029t 25741770

[bb35] Werner, J., Tomkowicz, Z., Rams, M., Ebbinghaus, S. G., Neumann, T. & Näther, C. (2015*b*). *Dalton Trans.* **44**, 14149–14158. 10.1039/c5dt01898f 26182402

[bb36] Westrip, S. P. (2010). *J. Appl. Cryst.* **43**, 920–925.

[bb37] Wöhlert, S., Jess, I. & Näther, C. (2013). *Inorg. Chim. Acta*, **407**, 243–251.

[bb38] Wöhlert, S., Tomkowicz, Z., Rams, M., Ebbinghaus, S. G., Fink, L., Schmidt, M. U. & Näther, C. (2014). *Inorg. Chem.* **53**, 8298–8310. 10.1021/ic500572p 25080077

[bb39] Yang, G., Zhang, Q., Zhang, X. P., Zhu, Y. & Ng, S. W. (2007). *J. Chem. Res.* pp. 384–386.

