# Supplementary figures and images for: Synthesis, crystal structure and thermal properties of bis­(aceto­nitrile-κN)bis­(3-bromo­pyridine-κN)bis­(thio­cyanato-κN)cobalt(II)
Source: Acta Crystallogr E Crystallogr Commun. 2023 Jan 1;79(Pt 1):14–8. doi: 10.1107/S2056989022011380 (PMC9815133; doi:10.1107/S2056989022011380)

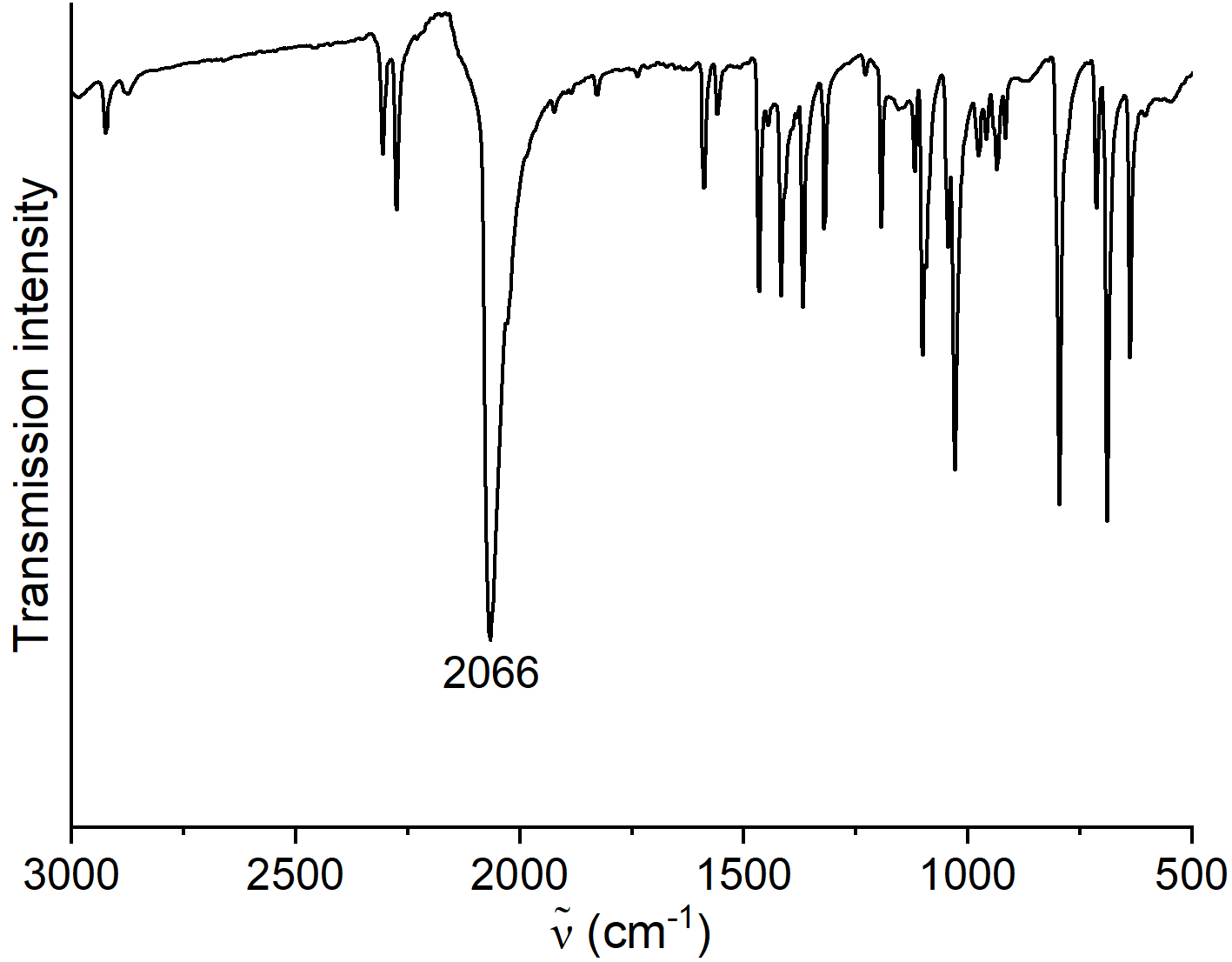

Supplement: Supplementary file 3 [file e-79-00014-sup3.png]

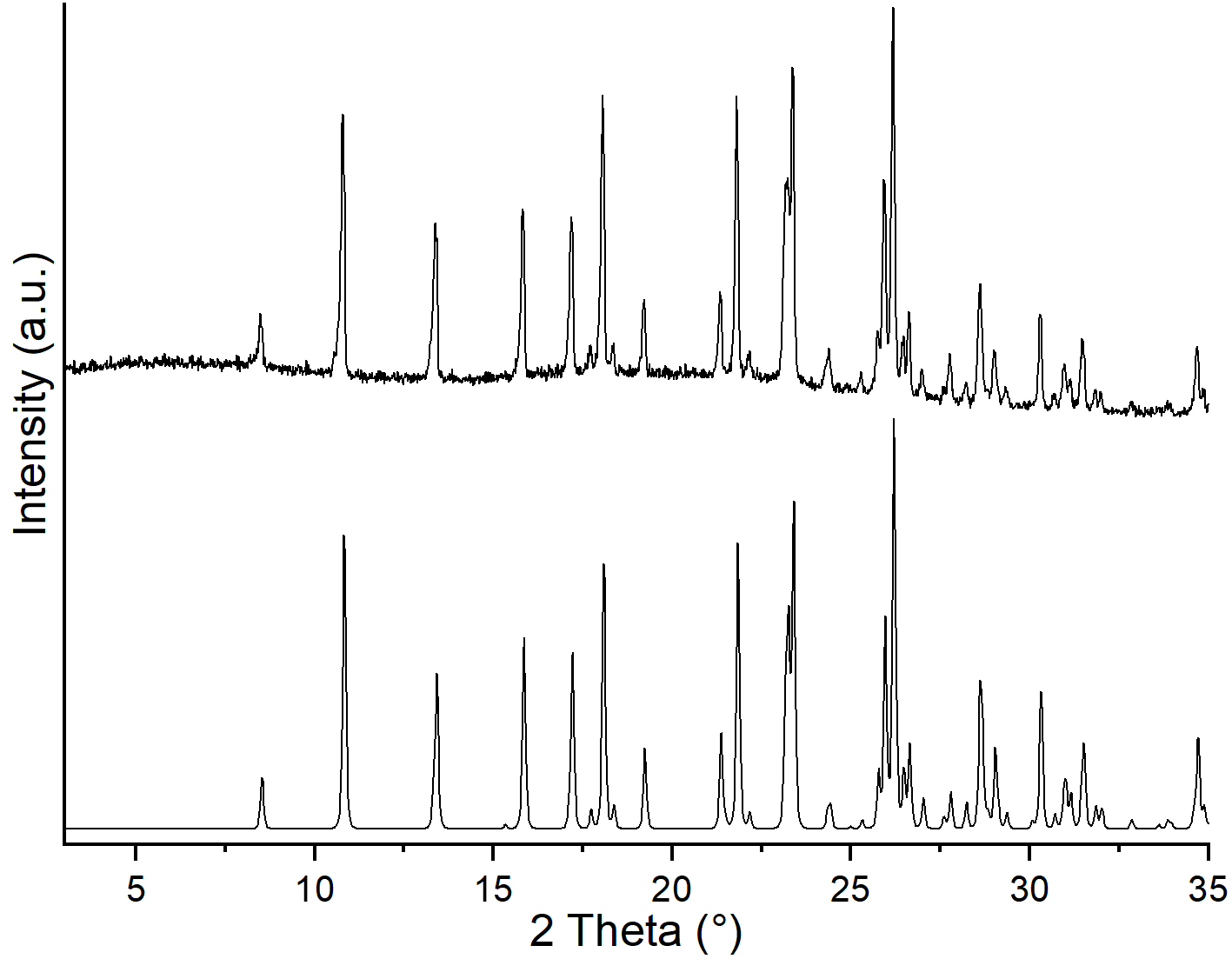

Supplement: Supplementary file 4 [file e-79-00014-sup4.png]

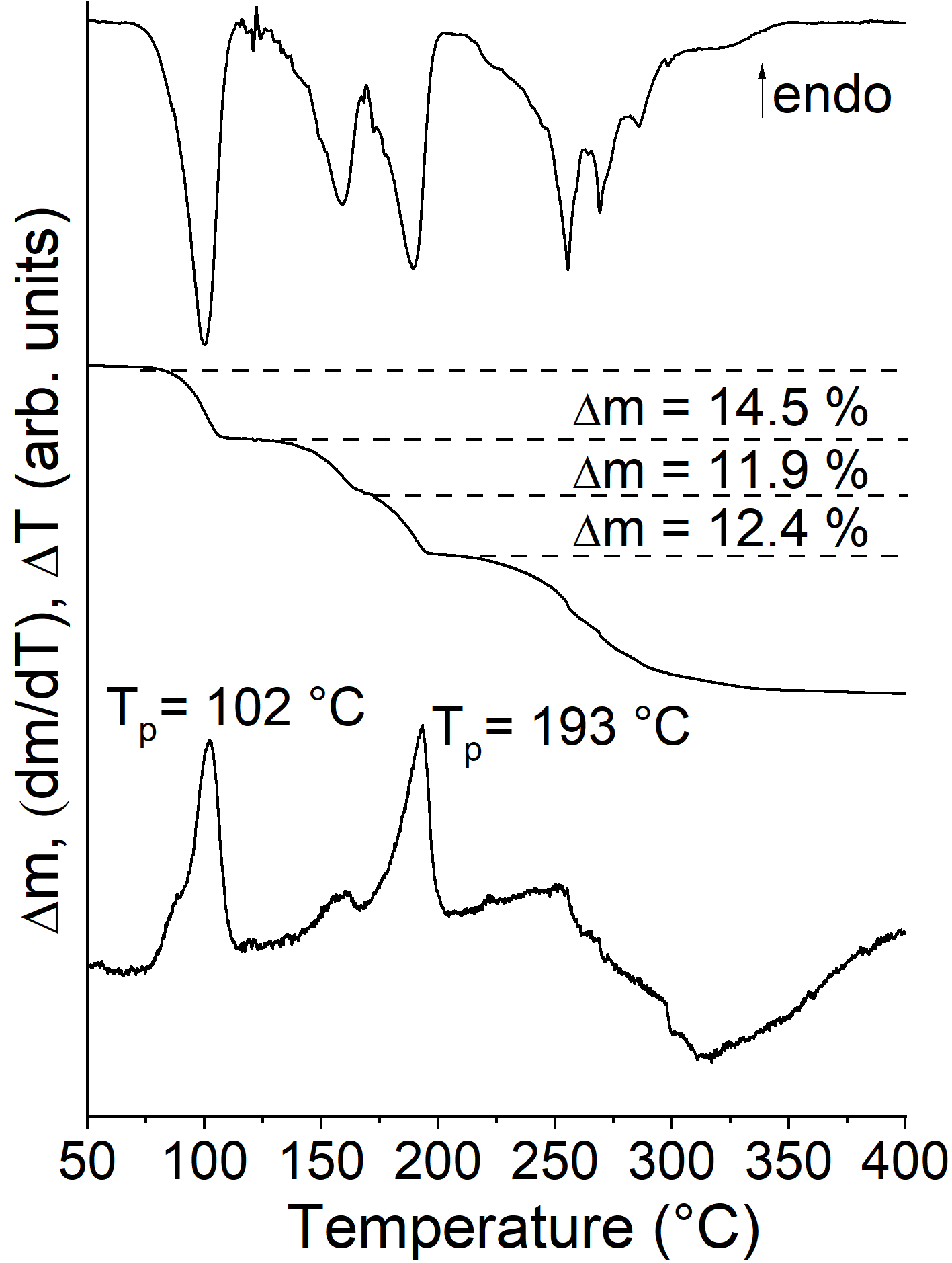

Supplement: Supplementary file 5 [file e-79-00014-sup5.png]

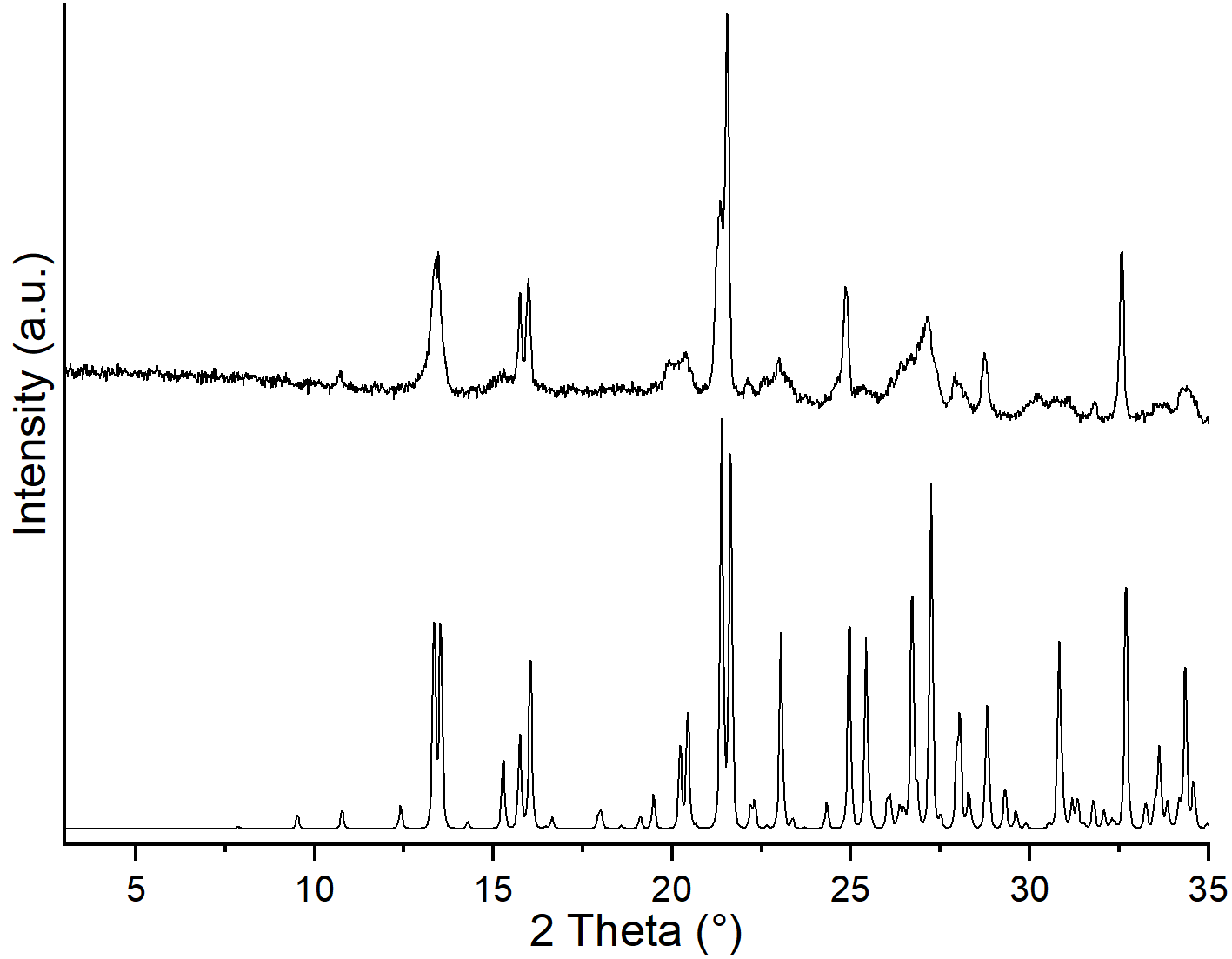

Supplement: Supplementary file 6 [file e-79-00014-sup6.png]
